# Morphometric Findings in Adolescents with Robin Sequence: A Photographic and Cephalometric Study of the Face and Mandible

**DOI:** 10.3390/children13020242

**Published:** 2026-02-09

**Authors:** Silvia Müller-Hagedorn, Helen So, Brigitte Vi-Fane, Véronique Soupre, Bachar Houssamo, Nancy Vegas, Walter Lehmacher, Arnaud Picard, Véronique Abadie

**Affiliations:** 1 Pediatric Maxillofacial and Plastic Surgery Unit, Center for Rare Diseases “Clefts and Facial Malformations”, Necker University Hospital, Assistance Publique-Hôpitaux de Paris (APHP), 149 Rue de Sèvres, F-75015 Paris, France; 2 Department of Orthodontics, Pitié Salpétrière University Hospital, Assistance Publique-Hôpitaux de Paris (APHP), Sorbonne University, UFR d’Odontologie, 47-83 Boulevard de l’Hôpital, F-75013 Paris, France; 3 General Pediatrics Unit and Referral Center for Rare Diseases “Syndrome de Pierre Robin et Troubles de Succion-Déglutition Congénitaux”, Necker University Hospital, Assistance Publique-Hôpitaux de Paris (APHP), 149 Rue de Sèvres, F-75015 Paris, France; 4 UFR de Médecine, Paris Cité University, 15 Rue des Ecoles, F-75006 Paris, France; 5 Institute of Medical Statistics and Epidemiology, University Hospital of Cologne, Kerpener Strasse 62, D-50937 Cologne, Germany

**Keywords:** Robin sequence, Stickler syndrome, maxillo-mandibular growth, radiographic cephalometry, adolescence, facial profile, Delaire’s analysis, cleft palate, facial morphology, obstructive sleep apnea

## Abstract

**Highlights:**

**What are the main findings?**

- Adolescents with Robin sequence mainly presented proportionate retrusion of both jaws, which was mostly associated with skeletal Class I and hyperdivergent facial patterns. In subjective profile analysis, approximately 84% of patients had good or acceptable profiles, with no major deficit of chin projection.
- During the growth spurt, the mandibles of patients with Robin sequence exhibited greater growth velocities than those of controls. Nevertheless, the mandibles remained harmoniously downsized. This finding is consistent with partial catch-up growth.

**What are the implications of the main findings?**

- The degree of neonatal retrognathia did not impact the sagittal skeletal mandibular parameters of adolescents, as shown by the results of the ANOVA. Therefore, neonatal retrognathia cannot be considered a prognostic factor for further mandibular growth. The facial divergence increased with the degree of neonatal functional impairment. This underscores the importance of functional co-treatment.
- Robin sequence seems to affect both jaws, and further research is needed to determine the full implications of this finding.
- The bi-retrognathic facial pattern is a risk factor for sleep-disordered breathing.

**Abstract:**

Background: The aims of the study were to describe facial morphology and analyze facial growth in adolescents with Robin sequence (RS) or Stickler syndrome. Methods: The facial morphology, mandibular size, and facial growth of 69 adolescents (ages 12–18) with RS were analyzed using existing cephalometric radiographs (*n* = 37) and photographs (*n* = 69). All participants were followed in our institution since birth. None underwent growth-modifying treatment for micrognathia during infancy, but all had conservative orthodontic treatment during adolescence. Results: Cross-sectional cephalometric analysis according to Tweed revealed differences in RS adolescents as compared with reference values, such as a proportionate retrusion of both jaws, as indicated by decreased SNA and SNB angles (*p* < 0.05). This finding was mostly associated with skeletal Class I (62.2%) and a vertical facial pattern as indicated by increased FMA and CoGoMe angles (*p* < 0.05). In Delaire’s analysis, patients showed decreased maxillary, maxillary alveolar (*p* < 0.05), and mandibular body territories (*p* > 0.05) but increased ramus (*p* > 0.05) and nasopremaxillary territories (*p* < 0.05). According to Ricketts’ analysis, mandibular width was decreased in half of our patients (*p* > 0.05). The mandibles were harmoniously downsized before and after the growth spurt (*p* < 0.05); however, they exhibited greater growth velocities than controls. A long-term study during puberty revealed an increase in SNB angles and a decrease in ANB angles (both *p* < 0.05), which improved the maxillomandibular relationship. Additionally, the vertical facial pattern attenuated (FMA, SNGoGn, and CoGoMe angles decreased; *p* > 0.05). On cross-sectional photographic analysis, 33.3% of patients had an orthofrontal (straight), 59.4% a cisfrontal (convex), and 7.3% a transfrontal (concave) profile. Their vertical facial proportions were normal. In the subjective profile analysis, most patients (approximately 84%) had good or acceptable profiles, with no major deficit of chin projection. The initial degree of neonatal retrognathia and type of cleft palate surgery did not affect major skeletal parameters (*p* > 0.05). However, the degree of neonatal functional impairment affected the vertical parameters (SNGoGn, FMA angle; *p* < 0.05). Conclusions: Overall, RS patients presented a bi-retrognathic profile, a normal jaw relationship, and a tendency toward a vertical growth pattern. Partial mandibular catch-up growth occurred during the pubertal growth spurt. The degree of neonatal retrognathia does not predict further mandibular growth.

## 1. Introduction

Robin sequence (RS) is considered a malformative triad characterized by mandibular micro- and retrognathia, glossoptosis, and upper airway obstruction. A U-shaped cleft palate (CP) is often present, though not mandatory [1]. Micro- and retrognathia are the suspected initial anomaly, which leads to glossoptosis, hindering the palatal shelves from fusing at the end of the second month of gestation. The posterior displacement of the tongue (glossoptosis), secondary to mandibular hypoplasia, causes varying degrees of upper airway obstruction in affected neonates [2,3]. Therefore, mandibular growth restriction is hypothesized to be the initiating event in RS and therefore an important etiologic factor for breathing disorders in these patients. RS occurs globally and affects 9.5 out of 100,000 live-born infants, depending on RS definition [4]. There is a trend toward higher birth prevalence in the European population. An epidemiological survey estimated a birth prevalence of 12.4 per 100,000 live births in Germany [5] and 14.0 per 100,000 live births in France [6].

Early treatment approaches vary depending on the severity of respiratory disorders and feeding problems as well as the habits and experiences of the medical team. These range from conservative treatments, such as prone positioning, continuous positive airway pressure (CPAP), high-flow nasal cannula, nasopharyngeal airway, Tuebingen Palatal Plate/Stanford Orthodontic Airway Plate, and nasogastric tube feeding [3,7,8,9], to more invasive surgical treatments, such as tongue–lip adhesion, mandibular distraction osteogenesis, and tracheostomy [10,11,12,13,14,15,16,17]. Some early treatment concepts rely on catch-up growth of the deficient mandible, whereas others directly increase size and promote growth of the deficient mandible.

RS is a heterogeneous entity, both clinically and pathogenetically [18]. It may occur as an isolated entity (isolated RS [iRS]), as a part of a syndrome, or associated with other malformations without any identified syndrome. Recently, an estimated 40% of RS cases were considered isolated, whereas 60% were syndromic or associated [19]. More than 100 syndromes have been documented as associated with RS. The most common include Stickler syndrome (34%), 22q11 microdeletion syndrome (11–15%), fetal alcohol syndrome (10%), and Treacher Collins syndrome (5%) [20,21]. In RS, deficient mandibular outgrowth resulting in reduced mandibular size may be attributed to intrinsic abnormalities (e.g., genetic influences) [22], extrinsic factors mainly related to mechanical restraints (e.g., oligohydramnios, multiple fetuses, and abnormal embryonic implantation) [23], or neurological factors (e.g., brain or brainstem anomalies), as well as neuromuscular diseases [24]. This heterogeneity may be a potential explanation for the observed variations in interindividual growth. A more profound understanding of the etiology and pathogenesis of mandibular development may allow for anticipating the potential mandibular development and creation of personalized treatment strategies.

The existing body of knowledge concerning craniofacial and mandibular morphology and growth in adolescents with RS is limited and partly contradictory: Suri et al. [25] reported that adolescents with iRS presented smaller cranial base lengths, shorter maxilla- and mandibular lengths, bimaxillary retrognathism, and mandibular deficiency, which was more pronounced in the mandibular body than the ramus, thus leading to mandibular retrognathia as compared with unaffected counterparts. Furthermore, patients with iRS exhibited a vertical growth pattern, increased mandibular plane inclination, and obtuse gonial angles. Shen et al. [26] compared patients with iRS during early (ages 4–7 years) and late (ages 10–13 years) childhood with age-matched patients with isolated CP (iCP). In early childhood, the mandibular length was comparable between both groups. However, in older patients (10–13 years), the mandibular length was significantly shorter in the iRS group, and they did not present a sagittal jaw discrepancy due to a proportionate deficiency of maxillary and mandibular lengths. Laitinen and Ranta [27] compared 10-year-old patients with iRS to patients with iCP. The main difference was that patients with iRS had a more retrusive mandible. However, during the 4-year follow-up period, the growth rates of the jaws did not differ between the two groups and the initial jaw relationship remained unchanged. Furthermore, Daskalogiannakis et al. [28] evaluated the mandibles of patients with iRS and iCP at age 6, 10, and 17 years. In patients with iRS, the mandibular length consistently remained 4% to 5% shorter at all ages and did not show any acceleration of growth.

The team from Necker Hospital (University of Paris Cité, France) recently reported on the satisfactory quality of life (QoL) of adolescents with RS [29]. Treatment has improved nowadays, and patients born with iRS no longer present neurological and cognitive sequelae resulting from neonatal airway obstruction [30,31]. Therefore, the current focus is on improving QoL, long-term function, and psychological results by addressing facial morphology and growth. Moreover, a more comprehensive understanding of facial growth is crucial for selecting the best possible and most effective treatment strategy for this patient group.

This retrospective, observational cohort study aimed to analyze the facial and mandibular morphology and growth of adolescents with iRS mainly or RS associated with Stickler syndrome. The study population was homogeneous and had been followed up since their neonatal period at our institution. Furthermore, we evaluated the impact of initial functional severity (e.g., feeding and breathing issues), the degree of neonatal retrognathia, and the effect of CP repair (one-step vs. two-step surgery) on facial and mandibular morphology and growth during adolescence. Additionally, we qualitatively assessed patients’ faces to determine whether the typical stigmata of RS were still detectable in affected adolescents. The main objective was to describe the typical facial patterns of adolescents with RS and report any deviations from the norm. This information will help improve treatment strategies for this distinctive patient group.

The research questions were as follows:

1. Do adolescents with RS have different facial morphology than healthy, unaffected controls (population represented by reference values for cephalometric and photographic assessment)?
2. Does the deficient mandible undergo catch-up growth during the pubertal growth spurt?
3. Do the following features affect skeletal parameters in cephalometric analysis: diagnosis (iRS vs. Stickler syndrome), type of surgery for palatal repair (one-step vs. two-step surgery), degree of neonatal retrognathia, and degree of neonatal functional impairment?
4. What are the results of the subjective evaluation of the facial profiles of adolescents with RS?

This study used a mixed-methods approach, combining quantitative and qualitative data originating from cephalometric and photographic assessments of the facial morphology and mandibles of adolescents with RS.

## 2. Materials and Methods

### 2.1. Study Population

This study, initiated between 2016 and 2019, identified all patients with RS born between 7 January 1997 and 7 January 2007, who were admitted to Necker or Trousseau Hospitals (University of Paris Cité, France) during the neonatal period. The age range at enrolment was 12 to 18 years (mean [SD] 14.4 [1.8] years).

The inclusion criteria were iRS, underlying Stickler syndrome, or RS in addition to other minor bone malformations without impact on neurocognitive development. Additionally, patients had to have undergone a full follow-up at our institution and given their consent to participate.

All patients who met these criteria (72 adolescents) were included in this study: 59 with iRS, nine with Stickler syndrome, and four with RS and minor bone anomalies, all of whom had CP. The diagnosis was confirmed by a geneticist. All patients had normal karyotype and Array CGH. In this series, 15 patients (20%) had a family history of Robin sequence. Of these, eight had iRS and seven had non-isolated RS (5 with Stickler syndromes and 2 with minor bone anomalies). The vast majority of patients were Caucasian (96%), while three patients (4%) were of another ethnic origin. Depending on the severity of breathing disorders, airway obstruction was primarily treated with prone positioning (62 patients), CPAP (1 patient), or tracheostomy (9 patients). None of the children underwent mandibular distraction osteogenesis, tongue–lip adhesion, or other growth-promoting treatments in infancy.

Palatal repair was performed either as a one-step procedure at age 9 months (uranostaphyloraphy via the Veau–Wardill technique [32] or intravelar veloplasty according to Sommerlad [33], combined with hard palate repair) or as a two-step procedure, with intravelar veloplasty according to Sommerlad [33], performed at age 6 to 8 months, followed by hard palatal closure 6 to 8 months later. The oral-specific QoL was assessed using the Child Oral Health Impact Profile (COHIP-SF 19) in the patient group. Scores for adolescents with iRS were comparable to those of unaffected controls [29]. Patients were evaluated by speech–language therapists during their annual checkups at Necker Hospital. If necessary, treatment was recommended. Various practitioners performed orthodontic treatments because patients received treatment close to home. Therefore, little was known about the orthodontic treatment they received. None of the patients underwent orthognathic surgery during the study period, but one had genioplasty at age 16. Patients were evaluated during their annual checkup at the Necker Hospital, the French national referral center for rare diseases in children with RS. They were subdivided according to their diagnosis (e.g., iRS versus Stickler syndrome); the functional severity of their feeding and breathing disorders at the time of initial diagnosis according to the Couly classification, as modified by Cole [34] (grade 1: prone position and feeding facilities; grade 2: prone position and gavage feeding; grade 3: airway intervention); the degree of their neonatal retrognathia (distance between alveolar ridges: minor <5 mm, moderate 5–9 mm, major >10 mm); and the type of surgery (one- versus two-step palatal repair).

### 2.2. Material

This study used a mixed-methods approach to evaluate lateral and frontal radiographic cephalograms as well as extraoral photographs.

#### 2.2.1. Radiographic Cephalograms

Patients were positioned in the cephalostat with their heads in a natural head position so that the Frankfort horizontal (FH) plane was oriented parallel to the floor. For lateral cephalograms, the midsagittal plane was perpendicular to the X-ray beam and parallel to the film plane. For the posteroanterior cephalogram, the patient was rotated 90° to face the film. The jaws were in occlusion, and the lips were gently closed.

Only cephalograms with correct patient positioning within the cephalostat were retained for analysis. Cephalometric analyses were performed using Ortholeader software (Ortholeader Couleur et Connection version 10.6, groupe Dentalsoft, Taluyers, France) and Delairecephalo software (Delairecephalo version 1.0.0, France) for Delaire analysis.

#### 2.2.2. Extraoral Photographs

A medical photographer took extraoral frontal and profile photographs of patients by using a Nikon Reflex D7000 camera (Nikon Corporation, Tokyo, Japan) equipped with a 60 mm macro lens and a ring flash. Photographs were taken at the time of inclusion in this study. The patient’s head was oriented according to the FH plane, with the eyes looking straight ahead and the ears uncovered. The lips were closed, and the jaws were in occlusion. The photographs were analyzed using Geogebra Geometry software (https://www.geogebra.org, accessed on 1 January 2019).

### 2.3. Procedures: Cephalometric and Photographic Analyses

#### 2.3.1. Cross-Sectional Cephalometric Analyses

**Figure 1:**
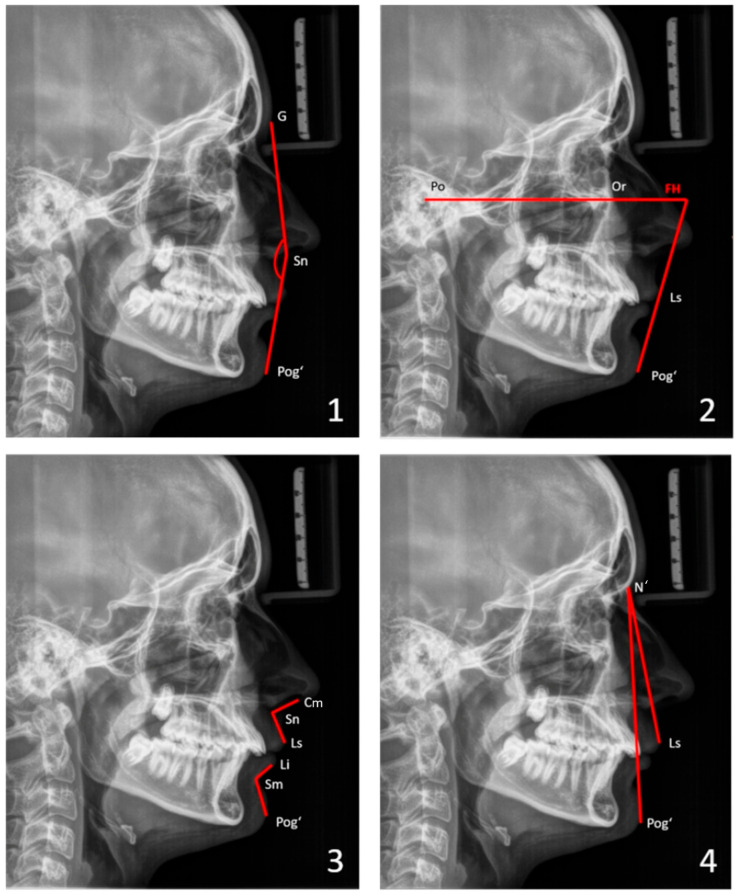
Soft-tissue analysis: (**1**) facial convexity angle (GSnPog‘), (**2**) Z angle (chin–lip profile line to Frankfort horizontal [FH] line), (**3**) nasolabial angle (CmSnLs) and mentolabial angle (LiSmPog‘), (**4**) chin projection (LsN‘Pog‘). See the Appendix A for the definitions of landmarks and angles.

For soft-tissue cephalometric analysis, the facial profile was evaluated by an angular analysis. Facial convexity (GSnPog’), Z angle (the convexity of the lower facial profile, which is the intersection of FH and a line connecting the Pog’ and the most prominent lip), nasolabial angle (CmSnLs), mentolabial angle (LiSmPog’), and chin projection (LsN’Pog’) were considered (Figure 1). See the Appendix A for the definitions of landmarks and angles.

**Figure 2:**
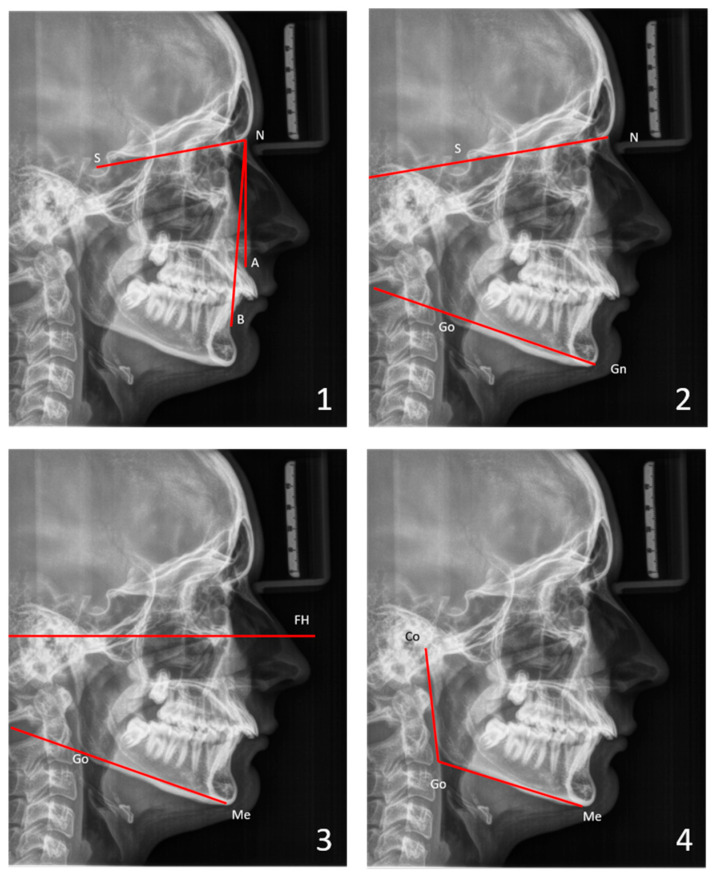
Skeletal analysis with sagittal and vertical parameters: (**1**) SNA, SNB, ANB angles; (**2**) facial plane divergence (SN-GoGn) angle; (**3**) FH plane-mandibular plane (FMA) angle; (**4**) gonial (CoGoMe) angle. See the Appendix A for definitions of landmarks and angles.

For the skeletal cephalometric analysis (a simplified analysis according to Tweed [35]), the sagittal positions of the maxilla (SNA) and the mandible (SNB) in relation to the cranial base, as well as their relative position to each other (ANB), were determined. Vertical parameters, such as the facial plane divergence (SN-GoGn angle), the FH plane–mandibular plane angle (FMA), and the gonial angle (CoGoMe), were considered to quantify the facial divergence (Figure 2). See the Appendix A for definitions of landmarks and angles.

**Figure 3:**
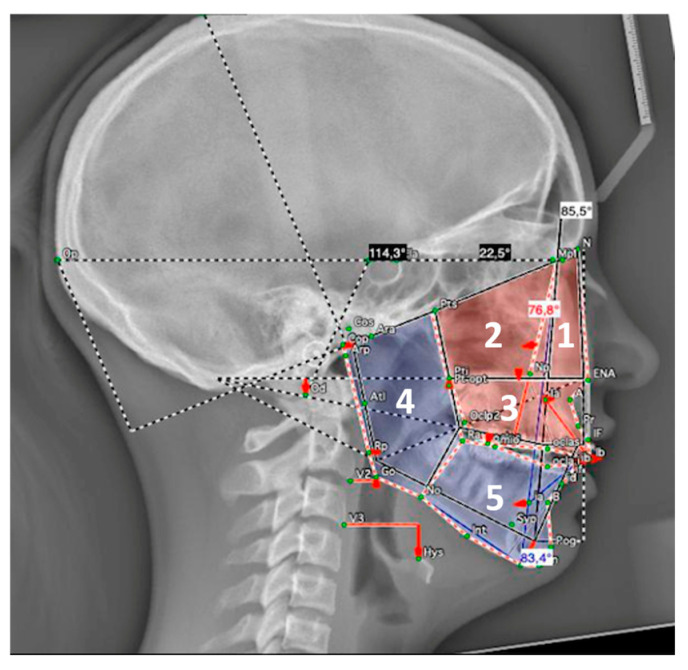
Analysis according to Delaire: 5 territories: nasopremaxillary (1), maxillary (2), maxillary alveolar (3), ramus (4), corpus (5). Colored areas: determined surfaces; dotted lines: ideal surfaces.

For the architectural analysis according to Delaire, only cephalometric radiographs with a scale were used. Topographic analysis compared the areas of the five distinctive architectural territories of the facial skull (nasopremaxillary, maxillary, maxillary alveolar, ramus, and corpus of the mandible) to patient-specific optimal areas (patient-specific ideal norms). These territories are anatomically defined by four cranial lines (C1-C4) and eight craniofacial lines (CF1-CF8) [36,37] (Figure 3).

**Figure 4:**
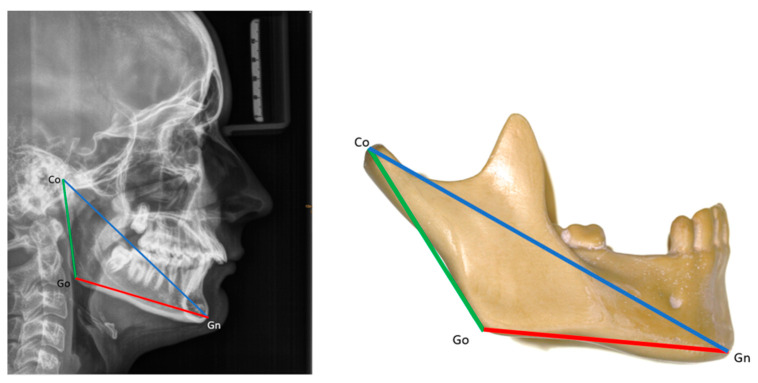
Mandibular size: mandibular length (Co-Gn), ramal length (Co-Go), body length (Go-Gn). See the Appendix A for definitions of landmarks.

Mandibular size was assessed according to Suri et al. [25]. The following measures were taken: total mandibular length (Co-Gn), ramal length (Co-Go), body length (Go-Gn), and the ramal-to-body length ratio (Co-Go/Go-Gn) (see the Appendix A for definitions of landmarks). Mandibular sizes were evaluated in patients in two age groups: 11–13 years old (age 1; mean age: 11.7 years) and 14–18 years old (age 2; mean age: 15.6 years). These results were then compared to those of age-matched non-RS individuals from the normative cephalometric collection of Caucasian patients at the Burlington Facial Growth Research Center, Faculty of Dentistry, University of Toronto (mean age 1: 11.8 years; mean age 2: 16.6 years) [25] (Figure 4).

**Figure 5:**
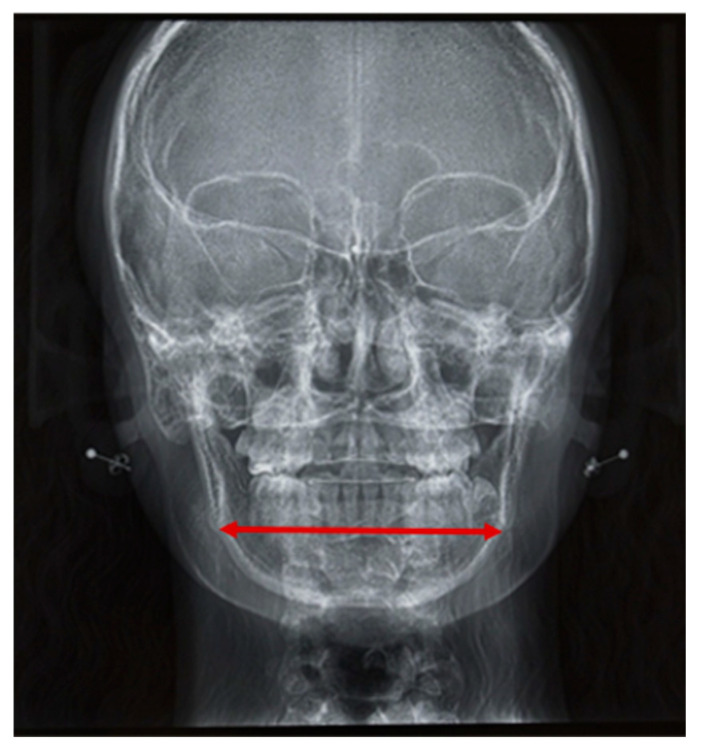
Determination of mandibular width on frontal radiographic cephalograms.

Mandibular width was determined by measuring the distance between the two antegonial notches on frontal cephalometric radiographs. Age-dependent reference values were used for this analysis (analysis according to Ricketts [38]) (Figure 5).

#### 2.3.2. Longitudinal Growth Study

Facial and mandibular growth was studied in patients who had two cephalometric radiographs taken with an interval of at least 2 years. Skeletal sagittal parameters (SNA, SNB, ANB angles) and vertical parameters, such as the facial plane divergence (SN-GoGn angle), the FH plane–mandibular plane angle (FMA), and the gonial angle (CoGoMe), were determined. Additionally, the Z angle from soft-tissue analysis was used to evaluate the convexity of the lower facial profile during growth (see the Appendix A for definitions).

#### 2.3.3. Photographic Analyses

**Figure 6:**
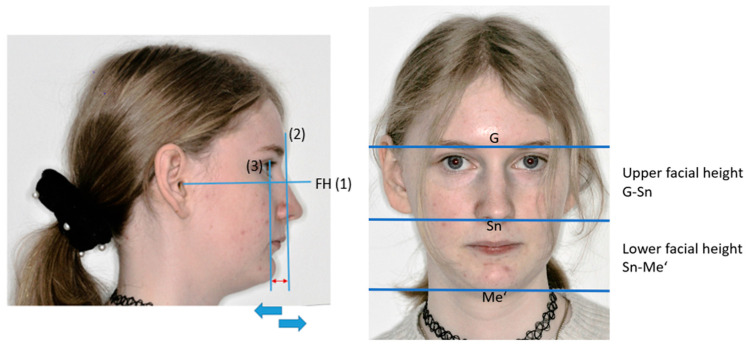
(**Left**) Subnasal profile: chin/lower lip within profile field (orthofrontal), behind (cisfrontal), ahead (transfrontal). The blue arrows indicate the different positions of the chin or lower lip: the blue arrow to the left side shows a cisfrontal/convex profile, while the blue arrow to the right shows a transfrontal/concave profile. (1) FH: Frankfort horizontal line; (2): soft-tissue glabella perpendicular; (3) orbital perpendicular; jaw profile field. (**Right**) Vertical facial proportions: upper facial height: glabella (G) to subnasal point (Sn); lower facial height: subnasal point (Sn) to soft-tissue menton (Me‘); total facial height: glabella (G) to soft-tissue menton (Me‘). (Photographs shown with consent of patient.) See the Appendix A for definitions.

The subnasal profile was evaluated according to the Izard and Simon classification [39], which grades the subnasal skin profile based on the position of the chin and the lower lip in relation to the jaw profile field. This classification is based on the construction of three reference lines: (1) the FH, (2) the soft-tissue glabella perpendicular, and (3) the orbital perpendicular. These two perpendicular lines define the jaw profile field. An orthofrontal (straight) profile, considered aesthetically pleasing, is defined by the position of the chin and the lower lip within the demarcated area. If the chin or lower lip is behind or ahead of the demarcated area, the profile is called cisfrontal (convex) or transfrontal (concave) (Figure 6). Data were collected on a nominal scale.

Vertical facial proportions are evaluated by the ratio of upper facial height (glabella [G] to subnasal point [Sn]) to total facial height ([G] to soft-tissue menton [Me’]) and lower facial height (Sn to Me’) to total facial height. This ratio is then compared with the 50%/50% ratio that indicates aesthetic balance (Figure 6). These ratios are determined with metric values. (See the Appendix A for definitions).

**Figure 7:**
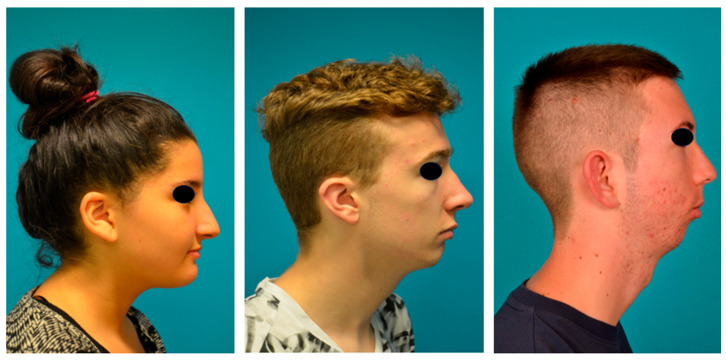
Patients exhibiting good, acceptable, and poor profiles (photographs shown with consent of parents and patients).

For the subjective evaluation of the facial profile, lateral photographs of the patients’ faces were assessed independently by a maxillofacial surgeon and a pediatrician (rater group 1) and by two orthodontists (rater group 2), all familiar with patients with RS. The criteria defined the question to be answered: Were the initial stigmata of RS (e.g., micrognathia, recessive mandible, convex profile, deficient chin projection, decreased distance between the chin and neck, and cervico-chin angulation defect) and the hyperdivergent facial pattern with an increased gonial angle still noticeable? Or was the profile aesthetically correct (straight), presenting none of the aforementioned features? Three profile types were defined: a good (aesthetically adequate) profile was generally straight, with normal vertical facial proportions. It showed no maxillary–mandibular discrepancy, and the chin projection and gonial angle were normal. The distance between the chin and the neck was normal, and there was no cervico-chin angulation defect. A poor profile was characterized by the opposite. An acceptable profile was in between (Figure 7). Next, the raters were blinded to all neonatal and surgical data and underwent a calibration protocol. In case of disagreement, consensus was obtained within the rater pair. In a subsequent step, the raters evaluated patients’ profiles.

#### 2.3.4. Subgroup Analyses

The impact of diagnosis (i.e., iRS vs. Sticker syndrome), one- vs. two-step surgery for palatal repair, degree of neonatal retrognathia, and degree of initial functional impairment on skeletal parameters were studied.

### 2.4. Reliability

Intra-rater reliability was tested by remeasuring all cephalometric and photographic parameters. The same experienced orthodontist remeasured the parameters of 10 randomly selected patients at a 4-week interval.

### 2.5. Statistical Analyses

This was a descriptive and exploratory study. The data were collected in Microsoft Excel and analyzed using IBM SPSS v22 (IBM, New York, USA). We interpreted 95% confidence intervals (CIs) and *p*-values descriptively. The normal distribution of the variables was checked with the Shapiro–Wilk test. A one-sample Student *t*-test (parametric) was used to compare measured and reference values, a one-sample Wilcoxon test for analyses according to Delaire, and a paired Wilcoxon test (non-parametric) for growth studies. A Wilcoxon–Mann–Whitney test was used to examine gender disparities in the subjective evaluation of facial profiles. One-way ANOVA (with the Levene test) was used to evaluate the impact of initial severity, and Student *t*-test to evaluate the impact of surgery on skeletal features. Cohen’s Kappa was used to measure inter-rater reliability. Intraclass correlation coefficients (ICCs) were calculated to analyze intra-observer consistency between two measurements. An ICC > 0.75 indicated good agreement between the initial and subsequent measurements. *p* < 0.05 was considered statistically significant.

## 3. Results

### 3.1. Patients

Overall, interpretable photographs were available for 69 patients (35 females). Of those, lateral cephalometric radiographs of sufficient quality were available for 37 patients (34 iRS and 3 Stickler syndrome) to be included in a cross-sectional study. Additionally, 17 patients had two radiographs taken at least 2 years apart (mean: 2.8 years) for inclusion in a longitudinal analysis. The radiographs were taken at two ages: T1 (ages 11–13 years; before the growth spurt) and T2 (ages 14–18 years; after the growth spurt).

Lateral cephalometric radiographs with a calibration scale were available for 29 patients. These radiographs were analyzed by cephalometric analysis according to Delaire [36,37], and mandibular size was evaluated according to Suri [25].

**Figure 8:**
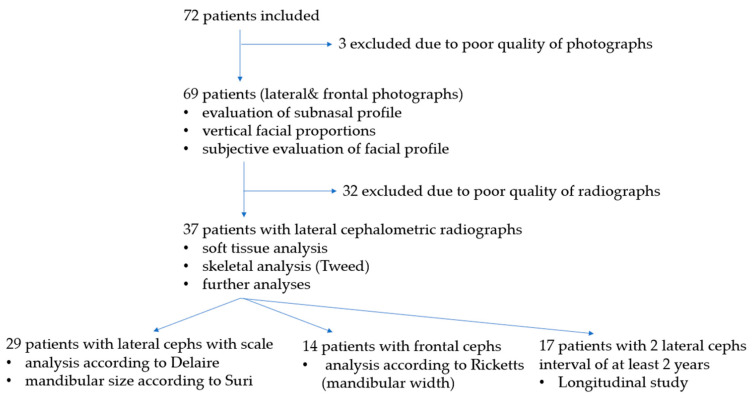
Flowchart of patients in this study.

For the latter analysis, the study group was divided into two subgroups: age 1 (*n* = 11), with radiographs taken when patients were 11–13 years old (mean: 11.7 years; before the growth spurt), and age 2 (*n* = 18), with radiographs taken when patients were 14–18 years old (mean: 15.6 years; after the growth spurt). Frontal cephalometric radiographs were available for 14 patients (Figure 8).

### 3.2. Intra-Rater Reliability

The ICCs ranged from 0.88 to 0.95 for all measurements (*p* < 0.05).

### 3.3. Results of Cephalometric Analyses

#### 3.3.1. Soft-Tissue Cephalometric Analysis (Table 1)

Facial convexity (GSnPog’) and the Z angle were slightly decreased as compared with reference values, confirming a tendency toward a convex profile. The nasolabial angle (CmSnLs) exhibited an increase, which might be indicative of a retrognathic maxilla. Additionally, an increased mentolabial angle (LiSmPog’) was noted. Chin projection (LsN’Pog’) was decreased and suggests a harmonious jaw relation. Taken together, these findings may suggest a bimaxillary retrognathism without substantial jaw discrepancy.

**Table 1:** Results of soft-tissue analysis (*n* = 37 patients). The downward arrow (↓) indicates a decrease in angle compared to the reference values, while the upward arrow (↑) indicates an increase.

Parameter Angle	Mean (Patients)	SD	Reference Value	Interpretation	*p* (*t*-Test)
Facial convexity GSnPog’	164.9°	5°	170°	↓	*p* < 0.001
Z angle	69.03°	5°	75°	↓	*p* = 0.001
Nasolabial angle CmSnLs	110.58°	8°	102°	↑	*p* < 0.001
Mentolabial angle LiSmPog’	142.3°	11.7°	122°	↑	*p* < 0.001
Chin projection LsN’Pog’	7.31°	2.31°	9.15°	↓	*p* < 0.001

#### 3.3.2. Skeletal Cephalometric Analysis According to Tweed [29] (Table 2)

Patients presented a retrognathic maxilla (SNA angle decreased) and a retrognathic mandible (SNB angle decreased). However, the jaw relationship was normal (ANB within reference values). Thus, our patient group exhibited a harmonious bimaxillary retrognathism. According to vertical skeletal analysis, patients exhibited a tendency toward a vertical (hyperdivergent) facial pattern due to increased SNGoGn, FMA, and CoGoMe angles.

**Table 2:** Skeletal sagittal and vertical features (*n* = 37 patients). Simplified analysis according to Tweed [35]. The downward arrow (↓) indicates a decrease in angle compared to the reference values, while the upward arrow (↑) indicates an increase.

Parameter Angle	Mean (Patients)	SD	Reference Value	Interpretation	*p* (*t*-Test)
SNA	76.40°	2°	82°	↓	*p* < 0.001
SNB	74.58°	2°	80°	↓	*p* < 0.001
ANB	1.84°	2°	2°	↓	*p* = 0.726
SNGoGn	37.70°	4.75°	36°	↑	*p* = 0.193
FMA	30.07°	3°	25°	↑	*p* < 0.001
CoGoMe	130.34°	5°	125°	↑	*p* < 0.001

FMA, Frankfort horizontal plane–mandibular plane angle.

Moreover, 29 patients (78%) exhibited a retrognathic maxilla, and only eight patients (22%) exhibited an orthognathic maxilla. A total of 29 patients (78%) had a retrognathic mandible, and twelve (41%) had an SNB angle <74°. Regarding the ANB angle, 23 patients (62%) had a skeletal Class I jaw relationship, six (16%) a Class II relationship, and eight (22%) a Class III relationship. Twelve patients (32%) presented a hyperdivergent pattern when considering the SNGoGn angle, seventeen (46%) had a normodivergent pattern, and only eight (22%) had a hypodivergent pattern. FMA angle was increased in 20 patients (54.1%), normal in twelve (32.4%), and decreased in five (13.5%). The gonial angle (CoGoMe) was increased in 21 patients (57%) and within the reference values in 16 (43%).

#### 3.3.3. Skeletal Cephalometric Analysis According to Delaire (Table 3)

The areas of the nasopremaxillary territory of patients exhibited an increase in area as compared to their patient-specific ideal norms. However, this increase occurred along with a substantial decrease in size of the maxillary territory. Taken together, the sum of these two differences was negative (−75,965 mm^2^). Nevertheless, the most significant deficit was in the maxillary alveolar territory. Therefore, the global maxillary surface area was less than the norm. Regarding the mandible, an increase in the area of the ramus was observed, along with a decrease in the area of the mandibular body. However, these differences did not reach statistical significance (*p* > 0.05).

**Table 3:** Comparison of observed and patient-specific ideal norms of growth territories (*n* = 29 patients).

Territory (mm^2^)	Mean Difference (Observed—Ideal)	SD	95% CI	*p* (Wilcoxon Test)
Nasopremaxillary	25.14	38.99	10.30 to 39.72	*p* = 0.002
Maxillary	−101.10	92.21	−136.18 to −66.03	*p* < 0.001
Maxillary alveolar	−246.10	203.47	−323.50 to −168.71	*p* < 0.001
Ramus	51.55	258.01	−46.59 to 149.70	*p* = 0.291
Corpus	−10.97	178.74	−78.95 to 57.02	*p* = 0.744

95% CI, 95% confidence interval.

#### 3.3.4. Mandibular Size (Table 4)

According to all measured parameters, the mandibles of patients with RS were significantly smaller than those of age-matched unaffected children, both before and after the pubertal growth spurt. However, RS patients demonstrated greater relative growth increments compared to unaffected children. Therefore, this observation may indicate a partial catch-up growth during the growth spurt. Additionally, the ramus exhibited a higher growth velocity than the body. Thus, the increase in total length was due more to the growth of the ramus than the growth of the body. Nevertheless, the ramus-to-body length ratio showed only a slight increase and did not differ from that of the reference group (*p* > 0.05).

**Table 4:** Patient and reference values * as well as growth increments (n_age1_ = 11 patients, n_age2_ = 18 patients).

	Mean (Patient)	SD	Range	Mean (Reference Values)	SD	*p* (*t*-Test)
Total length (mm)						
Co-Gn						
Age 1	96.61	9.23	(74.67–107.69)	114.11	4.81	*p* < 0.001
Age 2	108.00	9.24	(89.23–128.89)	123.17	6.81	*p* < 0.001
Growth increment	11.39 (+11.8%)			9.06 (+7.9%)		
Ramal length (mm)						
Co-Go						
Age 1	45.17	6.60	(33.85–52.31)	53.92	3.45	*p* = 0.001
Age 2	52.45	7.14	(43.08–66.67)	59.89	5.23	*p* < 0.001
Growth increment	7.28 (+16%)			5.97 (+11%)		
Body length (mm)						
Go-Gn						
Age 1	62.09	6.66	(45.33–68.57)	75.00	3.54	*p* < 0.001
Age 2	67.96	5.88	(55.38–80.00)	80.99	5.29	*p* < 0.001
Growth increment	5.87 (+9.5%)			5.99 (+8.0%)		
Ratio of						
ramal-to-body length						
Age 1	0.73	0.095	(0.52–0.82)	0.72	0.042	*p* = 0.734
Age 2	0.77	0.089	0.64–0.95	0.74	0.056	*p* = 0.135

Patients: age 1: 11–13 years (mean: 11.7), age 2: 14–18 years (mean: 15.6). Reference values: mean age 1: 11.8 years and mean age 2: 16.6 years. * From a cephalometric collection of Caucasian patients at the Burlington Facial Growth Research Center; Faculty of Dentistry, University of Toronto, Canada) [25].

#### 3.3.5. Mandibular Width

Mandibular width was inferior to the norm in half of our patient group (*n* = 14). However, the median difference between the measured values and the age-specific norms (according to Ricketts [38]) was −2.0 mm. Thus, patients showed a tendency toward a decreased distance between the two antegonial notches, although it was not significant (*p* = 0.821). Two patients with Sticker syndrome were included in this group. The differences between their determined values and the age-specific reference values were below the median for the entire patient group (−5.6 mm and −3.25 mm, respectively). Their mandibles were narrower than those of iRS patients.

#### 3.3.6. Results of the Longitudinal Growth Study (Table 5)

A total of 17 patients (all iRS) had two lateral cephalometric radiographs taken with an interval of at least 2 years (mean: 2.8 years). Patients in both age groups presented bi-retrognathic and vertical (hyperdivergent) facial patterns. Only the SNB and ANB angles differed significantly at T1 (before the growth spurt, at ages 11–13 years) and at T2 (after the growth spurt, at ages 14–18 years). The SNB angle increased by 1 degree, and the ANB angle decreased by approximately 1 degree (*p* < 0.05 for both), whereas the SNA angle remained unchanged (*p* = 0.92). The increase in the SNB angle during the growth spurt improved the jaw relationship. This finding was paralleled by a slight increase in the Z angle, though it was not statistically significant (*p* = 0.477). Furthermore, there was a tendency toward a normalization of the vertical excess (FMA angle decreased), though this did not attain statistical significance (*p* = 0.064).

**Table 5:** Results of long-term analysis (*n* = 17 patients).

Parameter	Mean T1 11–13 Years	Mean T2 14–18 Years	Mean Difference (T2-T1)	95% CI	*p* (Wilcoxon Test)
SNA	76.88°	77.00°	0.12	−0.86 to 1.09	*p* = 0.915
SNB	72.94°	73.94°	1.00	−0.26 to 2.26	*p* = 0.044
ANB	3.74°	2.82°	−0.91	−1.78 to 0.04	*p* = 0.007
Z angle	64.47°	65.94°	1.47	−2.89 to 5.83	*p* = 0.477
SNGoGn	38.50°	38.24°	−0.27	−2.04 to 1.51	*p* = 0.531
FMA	32.0°	30.0°	−2	−4.07 to 0.07	*p* = 0.064
CoGoMe	132.59°	132.29°	−0.29	−2.16 to 1.57	*p* = 0.887

95% CI, 95% confidence interval.

### 3.4. Results of Photographic Analyses (n = 69)

#### 3.4.1. Subnasal Profile

A total of 23 patients (33.3%) had an orthofrontal (straight) profile, and 41 (59.4%) had a cisfrontal (convex) profile to a lesser or greater degree. Only five patients (7.3%) presented a transfrontal (concave) profile.

#### 3.4.2. Vertical Facial Proportions

The ratio of upper facial height to total facial height and lower facial height to total facial height was 49.7%/50.3% overall and thus not different from the ratio of the healthy population (50%/50%) (*t*-test: *p* > 0.05). In patients with Stickler syndrome, this finding was slightly more pronounced: 48.7%/52% (*p* > 0.05, *n* = 7). The latter presented a slight tendency toward a vertical facial pattern.

#### 3.4.3. Subjective Evaluation of Facial Profile (Table 6)

Overall, rater group 1 judged 48% of profiles as good, and rater group 2 judged only 29% as such. According to rater group 1, approximately 38% of patients had acceptable profiles, whereas rater group 2 found that 54% of patients had acceptable profiles. Thus, most patients (approximately 84%) had a good or acceptable profile. Fewer than 17% of patients had poor profiles, according to rater groups 1 and 2 (Figure 8). In general, orthodontists were more critical in their assessments. Cohen’s kappa was 0.36, indicating fair agreement between the two groups of raters. The results of Wilcoxon–Mann–Whitney test showed no gender disparities (*p* = 0.1291 for rater group 1 and *p* = 0.4332 for rater group 2). Nevertheless, an obvious trend emerged: females’ facial profiles were rated more positively.

**Table 6:** Subjective assessment of facial profiles by different raters (contingency table). Gender differences are provided for males and females (M/F).

		Rater Group 2: Orthodontists			Total
		Good	Acceptable	Poor	
**Rater group 1: maxillofacial surgeon and pediatrician**	**Good**	**17** (6 M/11 F)	14 (7 M/7 F)	2 (0 M/2 F)	33 (13 M/20 F)
	**Acceptable**	2 (0 M/2 F)	**19** (13 M/6 F)	5 (2 M/3 F)	26 (15 M/11 F)
	**Poor**	1 (1 M/0 F)	4 (2 M/2 F)	**5** (3 M/2 F)	10 (6 M/4 F)
**Total**		20 (7 M/13 F)	37 (22 M/15 F)	12 (5 M/7 F)	69 (34 M/35 F)

### 3.5. Results of Subgroup Analyses

#### 3.5.1. iRS Versus Stickler Syndrome

Of the 37 patients who underwent skeletal analysis with lateral cephalometric radiographs, only three presented Stickler syndrome. Those with Stickler syndrome exhibited more pronounced retrusion of both jaws, particularly the maxilla, than those with iRS. This resulted in midfacial hypoplasia and a tendency toward skeletal Class III. For patients with Stickler syndrome, the mean SNA angle (absolute value 73.3°) was below the 20th percentile, the mean SNB angle (absolute value 73.3°) was below the 40th percentile, the mean ANB angle (absolute value 0°) was at the 20th percentile, and the mean Z angle (absolute value 72.0°) was above the 60th percentile for patients with iRS.

Additionally, these patients with Stickler syndrome exhibited a more vertical (hyperdivergent) facial pattern. The mean SNGoGn angle (absolute value 42.33°) was above the 75th percentile, the mean FMA angle (absolute value 31.3°) was above the 60th percentile, and the mean CoGoMe angle (absolute value 133.5°) was above the 75th percentile for patients with iRS. Furthermore, all patients with Stickler syndrome underwent a one-step palatal repair. According to the degree of neonatal retrognathia, two had severe retrognathia (≥10 mm), and one had moderate retrognathia (5–9 mm). Two patients had grade III neonatal functional impairment and required airway intervention, whereas one patient had grade II functional impairment and required gavage feeding. This subgroup exhibited worse values than patients with iRS for all studied parameters.

#### 3.5.2. One-Step Versus Two-Step Surgery for Palatal Repair

Of the 37 adolescents who underwent cephalometric analysis, 28 had one-step surgery for palatal repair and seven had two-step surgery; no information was available for two patients (*n* = 35). A Student’s *t*-test revealed that the type of surgery did not affect skeletal sagittal and vertical features, including the following angles: SNA (*p* = 0.991), SNB (*p* = 0.768), ANB (*p* = 0.706), SNGoGn (*p* = 0.332), FMA (*p* = 0.262), and CoGoMe (*p* = 0.853). Additionally, the type of surgery did not affect the areas of the nasopremaxillary (*p* = 0.384), the maxillary (*p* = 0.495), or the maxillary alveolar (*p* = 0.554) territories.

#### 3.5.3. Degree of Neonatal Retrognathia

Of the 37 patients who underwent skeletal cephalometric analysis, nine had minor retrognathia, 15 moderate retrognathia, and eleven major retrognathia at birth. Information was unavailable for two patients (*n* = 35). ANOVA revealed that only the CoGoMe angle (*p* = 0.01) was affected by the degree of neonatal retrognathia. The CoGoMe angle increased as the degree of neonatal retrognathia increased. The SNA (*p* = 0.815), SNB (*p* = 0.249), ANB (*p* = 0.164), SNGoGn (*p* = 0.134), and FMA (*p* = 0.171) angles were unaffected. Furthermore, neonatal retrognathia did not affect mandibular parameters, such as total mandibular length (age 1: *p* = 0.650/age 2: *p* = 0.525), ramal length (age 1: *p* = 0.729/age 2: *p* = 0.862), or body length (age 1: *p* = 0.345/age 2: *p* = 0.552), in patients before or after a growth spurt. In contrast, neonatal retrognathia affected only the maxillary territory (*p* = 0.019) in adolescents. The most important deficit of the maxillary territory was in patients with a low degree of neonatal retrognathia.

#### 3.5.4. Degree of Neonatal Functional Impairment

Among the 37 patients who underwent skeletal cephalometric analysis, twelve had grade I functional impairment, 16 had grade II functional impairment, and seven had grade III functional impairment at the time of initial diagnosis. For two patients, no information was available (*n* = 35). ANOVA revealed that the degree of initial functional impairment affected only skeletal vertical features, such as SNGoGn angle (*p* = 0.04) and FMA angle (*p* = 0.04). Both factors increased with degree of functional impairment, resulting in a more vertical (hyperdivergent) facial pattern. However, the SNA (*p* = 0.947), SNB (*p* = 0.297), ANB angles (*p* = 0.155), and CoGoMe (*p* = 0.083) angles remained unaffected. Additionally, the degree of functional impairment did not affect mandibular features, such as total mandibular length (age 1: *p* = 0.779/age 2: *p* = 0.678), ramal length (age 1: *p* = 0.836/age 2: *p* = 0.666), or body length (age 1: *p* = 0.445/age 2: *p* = 0.486), before or after the growth spurt.

### 3.6. Summary of Results (Table 7)

A summary of the results is presented in Table 7.

**Table 7:** Summary of results.

**Method**	**Major Results**
Soft-tissue cephalometrics	Suggests an underlying bimaxillary retrognathism without substantial jaw discrepancy and a slightly convex profile.
Skeletal cephalometrics (Tweed)	Bimaxillary retrognathism without jaw discrepancy, vertical (hyperdivergent) facial pattern (*p* < 0.05). 62% skeletal Class I, 16% Class II, 22% Class III.
Cephalometric analysis (Delaire)	Both maxillary territories decreased (*p* < 0.05). Territory of corpus decreased and ramus increased (*p* > 0.05 each).
Cephalometric assessment of mandibular size (Suri)	Total, ramal, body length harmoniously decreased (before and after pubertal growth spurt, *p* < 0.05). Increased growth velocities during growth spurt (partial catch-up growth).
Cephalometric assessment of mandibular width (Ricketts)	Decreased (*p* > 0.05).
Cephalometric growth study	Improvement of SNB and ANB (*p* < 0.05) but remaining harmonious bi-retrognathic pattern and a trend toward normalization of the hyperdivergent pattern (*p* > 0.05).
Photographic assessment of subnasal profile	Orthofrontal: 33.3%, cisfrontal: 59.4%, transfrontal: 7.3%.
Photographic assessment of vertical facial proportions	Normal: 49.7%/50.3% (*p* > 0.05%).
Subjective evaluation facial profile (photographs)	Approximately 84% with good/acceptable profile.
**Subgroup analysis (cephalometric parameters):**	
RS vs. Stickler syndrome	More pronounced bimaxillary retrusion, midfacial hypoplasia, tendency toward skeletal Class III in Stickler syndrome.
1-step vs. 2-step palatal repair	No impact on skeletal parameters (*p* > 0.05).
Degree of neonatal retrognathia	No impact on major skeletal parameters (SNA, SNB, ANB, and mandibular sizes; *p* > 0.05).
Degree of neonatal functional impairment	Facial divergence (SNGoGn and FMA) increased with degree of neonatal functional impairment (*p* < 0.05).

## 4. Discussion

Our study focused on the morphological outcomes, facial appearance, and growth trajectories of the face in adolescents with iRS or Stickler syndrome who were admitted to our institution during the neonatal period and monitored throughout adolescence. We used a mixed-methods approach to cross-validate the information obtained from various evaluation methods, such as cephalometric X-rays and photographs. This approach also mitigated the limitations of a single method.

We selected standard orthodontic landmarks that are easily identifiable and part of the standard orthodontic repertoire to provide a comprehensive view of the adolescents’ craniofacial morphology and mandibular size. An experienced orthodontist performed all analyses, achieving a high level of reliability.

### 4.1. Cephalometric Analyses

Soft-tissue cephalometrics revealed an underlying skeletal bimaxillary retrognathic facial pattern, which is consistent with the results of our skeletal analysis. However, our results should be interpreted with caution because the position and inclination of the incisors, as well as the thickness of the soft tissues, may affect the soft-tissue morphology. Nevertheless, significant correlations have been demonstrated between skeletal and soft-tissue evaluations [40,41]. Therefore, soft-tissue analysis confirms the results of the skeletal analysis.

Our cross-sectional, skeletal, cephalometric analyses revealed that most adolescents presented bimaxillary retrognathia and shorter mandibular lengths, although their jaw relationships were normal. Additionally, they exhibited slight vertical facial patterns. These results are consistent with those of Do et al. [42], who noted bimaxillary retrognathia and a hyperdivergent facial pattern in preadolescents with RS, as well as with those of Laitinen et al. [43], who observed comparable outcomes among young adults. Furthermore, our results align with the findings of Suri et al. [25], who reported that patients with iRS who underwent orthodontic treatment exhibited bimaxillary retrognathism, shorter maxillary and mandibular lengths, and a reduced cranial base length. Additionally, they exhibited an increased mandibular plane inclination, an obtuse gonial angle, and a vertical growth pattern as compared with controls. However, SNA and SNB are related to a reduced anterior cranial base in patients with RS. Thus, the severity of bimaxillary retrognathism in our patient group remains underestimated. Patients with severe skeletal anomalies and poor profiles will be recommended for orthognathic surgery once they have finished growing. This distinctive facial pattern is considered a risk factor for obstructive sleep apnea and predisposes this patient group to this condition [44,45,46]. In the literature, this is supported by cephalometric studies reporting decreased sagittal pharyngeal depth in patients with RS, thus indicating an ongoing risk for upper airway obstruction in this patient group [43]. For patients with decreased posterior airway space and obstructive sleep apnea, maxillomandibular surgical advancement can expand the airway.

Additionally, 62% of our patients had skeletal Class I, 16% skeletal Class II, and 22% skeletal Class III. A similar result was reported by Mendes Semensato et al. [46]. In Europe, the prevalence of Class I, Class II, and Class III in permanent dentition is 60.4%, 33.5%, and 6.2%, respectively [47]. We observed a higher proportion of patients with skeletal Class III in our patient group. These patients should be identified and monitored early on. They should receive comprehensive orthodontic treatment, as they will likely require orthognathic surgery at the end of their growth period. Furthermore, treatment modalities that pose a risk of inducing skeletal Class III should be questioned in this context.

Shen et al. [26] found that during late childhood (ages 10–13 years), the mandibular length was significantly shorter in patients with RS than in those with iCP. Additionally, their study revealed that patients with RS exhibited proportionated retrusion of both jaws rather than a sagittal jaw discrepancy. We confirmed these findings. Our patients also presented a proportionated retrusion of both jaws. When evaluating mandibular lengths, we found that our patients had smaller mandibles in all dimensions (total length, ramal length, body length) before and after the growth spurt as compared with reference values from the Burlington Growth Study [25].

When considering mandibular growth, Laitinen and Ranta [27] described a more receded mandible in 10-year-olds with RS than those with iCP. In addition, during the 4-year follow-up, the authors did not show an improvement in the jaw relationship of RS patients. In our study, we confirmed the first result: our patients also presented a retrognathic mandible. However, our longitudinal study demonstrated some normalization of the jaw relationship during the growth spurt. The SNB, ANB, and Z angles improved. Notably, our patients were older (14–18 years old) when they exhibited greater mandibular growth velocity.

Furthermore, we confirm the finding of Siqueira et al. [48], who reported an improvement in facial convexity with growth in a photographic study.

In a study by Daskalogiannakis et al. [28], mandibles were significantly smaller in adolescents with RS than in those with iCP, and no acceleration of growth was observed after the age of 5 years. We agree with them on one point, which is that the mandibles of our adolescents remained significantly smaller after the growth spurt as compared to the reference values from the Burlington Growth Study [25]. Additionally, the mandibles of our patients were proportionately downsized in all dimensions in that the ramus-to-body length ratio did not differ from that of the control group before or after the growth spurt. However, in contrast to Daskalogiannakis et al. [28], we demonstrated that our patients with RS experienced greater growth increments during the growth spurt than unaffected individuals. This finding may be considered a partial catch-up growth in adolescents. Thus, the mandibles of patients with RS may exhibit a twofold catch-up growth, the first occurring during infancy [49,50,51], followed by a second, partial one taking place during pubertal growth spurt.

According to Delaire’s analysis [36,37], we related the areas of the five different territories to patient-specific reference values. Unlike conventional cephalometric analysis, with reference values originating from unaffected individuals, Delaire’s analysis figures out reference values based on the skull structure of the individual in question. In this series, the areas of maxillary and maxillary alveolar territories were significantly smaller than expected given the size of skull. However, the area of the nasopremaxillary segment was larger than expected. Whether this finding can be attributed to compensatory growth remains unclear. In summary, the total maxillary area was reduced as compared with reference values. CP repair may be partly responsible for the restricted growth of the maxilla because of a reduced amount of bone related to the bony cleft and periosteal scarring. Regarding the mandible, the areas of the ramus were increased and those of the corpus decreased, although neither change was statistically significant. These observations suggest that RS affects both jaws. The decreased areas of corpus and maxillary alveolar territories align with clinical findings because patients with RS often exhibit dental crowding in both jaws and smaller upper and lower dental arches [52,53]. However, our analysis, based on Delaire’s methodology revealed a nonsignificant decrease in the mandibular body and an increase in the ramus. Conversely, our mandibular analysis, according to Suri, demonstrated significant differences between the determined values and reference values of mandibular parameters. We hypothesized that the entire skull structure might be affected in RS, thereby influencing patient-specific reference values.

This growth deficit in both jaws may be related to persisting tongue dysfunction, posterior tongue positioning, and hypotonia. Consequently, the tongue does not stimulate adequate growth in either jaw. This underscores the need for functional co-treatment. Improving the tongue’s function, tone, and position may promote growth in both jaws.

### 4.2. Photographic Analyses and Subjective Evaluation of Facial Profiles

According to the subnasal profile, 33.3% of our patients presented an orthofrontal (straight) profile, 59.4% a cisfrontal (convex) profile, and only 7.3% a transfrontal (concave) profile. Thus, approximately one-third of our adolescents with RS exhibited an aesthetically pleasing facial profile. We hypothesize that this proportion may increase during adolescence due to the increased growth velocity of the mandible. When we evaluated facial proportions, they did not differ significantly from those in the normal population. Therefore, our cohort exhibited vertically well-proportioned faces in photographs. The facial profiles were assessed subjectively by a pediatrician, a maxillofacial surgeon, and two orthodontists. Most patients (84%) were considered to have good or acceptable facial profiles, primarily attributed to good or acceptable chin projection. Of the patients with poor profiles, one had already undergone genioplasty to improve his profile. At the end of growth, orthognathic surgery will be recommended to the other patients with poor profiles and underlying severe skeletal disharmony. The two groups of raters showed fair agreement in their ratings. However, the orthodontists were more critical, perhaps because they are more familiar with this type of assessment. We could not find significant differences in the ratings of males and females, but we did find a tendency for females’ faces to be rated more positively. One possible explanation is that male faces in RS appear to have less pronounced jawlines. This could be linked to a lack of prominent male facial characteristics, which results in lower ratings. Nevertheless, when considered in their totality, these findings can be interpreted as a satisfactory result. Furthermore, healthcare professionals assigned lower facial appearance ratings to patients with a repaired lip and palate than laypeople [54]. Therefore, healthcare professionals and laypeople may have different perceptions of facial appearance and aesthetics. Consequently, it is plausible that laypeople may have assigned more favorable ratings to the faces of our patient group, thereby improving our results.

### 4.3. Subgroups

#### 4.3.1. Impact of Diagnosis: Stickler Syndrome Versus iRS

In our study, only three adolescents with Stickler syndrome underwent cephalometric analysis. As compared with patients with iRS, these patients exhibited a more pronounced vertical facial pattern and bimaxillary retrognathism. Specifically, their mean SNA and SNB angles were below the 20th and 40th percentiles, respectively, as compared with iRS patients. This situation was associated with midfacial hypoplasia. The mean ANB angle was 0°, indicating skeletal Class III. However, because of the small number of patients with Stickler syndrome in our cohort, these findings cannot be generalized. We can only report a tendency. However, in contrast to our results, Acke et al. [55] reported normal sagittal positions of the maxilla and mandible relative to the cranial base, as well as a normal maxillomandibular relationship.

Furthermore, we found that patients with Stickler syndrome were more likely to experience severe breathing and feeding difficulties after birth than iRS patients, so they were more prone to airway intervention. They exhibited a higher degree of neonatal retrognathia: two infants with grade III and one infant with grade II. Therefore, we recommend monitoring this subgroup more closely and providing more intensive treatment.

#### 4.3.2. Impact of One-Step Versus Two-Step Surgery for Palatal Repair

All our patients presented CP because CP was part of the RS diagnosis in our institution at that time. Therefore, whether the maxillary deficiency is attributed to RS or to CP repair remains unclear. The type of surgery did not seem to affect skeletal parameters. We expected to see a more pronounced growth deficit in patients who underwent one-step surgery for palatal repair. However, we found no link between skeletal parameters and width of the cleft, represented by the number of surgical steps. Thus, a small maxilla is likely partly intrinsic to the syndrome. Therefore, one-step surgery for palatal repair can be recommended. Further studies comparing patients with RS and iCP are needed to determine whether maxillary retrognathism is related to RS or to CP repair.

#### 4.3.3. Impact of Degree of Neonatal Retrognathia

The degree of neonatal retrognathia did not affect the skeletal parameters of our patients’ skeletal facial parameters except for the CoGoMe angle. This angle increased in parallel with the degree of neonatal retrognathia, resulting in a more vertical facial pattern. Of note, the degree of neonatal retrognathia did not affect the total, ramal, or body length of the mandible in adolescents, nor did it affect other skeletal parameters. According to Delaire’s analysis, neonatal retrognathia did not affect the mandibular territories, but it did affect the area of the maxillary territory. The maxillary territory exhibited the greatest deficit in adolescents, with a grade I degree of neonatal retrognathia. This observation may be linked to lingual dysfunction. These patients may have received only minimal therapy because they appeared inconspicuous as compared with their more severely affected counterparts. Therefore, we recommend treating all patients with RS, regardless of the degree of neonatal retrognathia. In conclusion, the degree of neonatal retrognathia cannot be considered a prognostic factor for further skeletal sagittal mandibular growth.

#### 4.3.4. Impact of Degree of Neonatal Functional Impairment

Initial functional impairment affected vertical skeletal parameters, such as the SNGoGn and FMA angles. These angles increased in parallel with the degree of functional severity, resulting in a more vertical (hyperdivergent) facial pattern. Therefore, functional treatment is mandatory to improve oral function and counteract a vertical facial pattern.

#### 4.3.5. Recommendations for General Dentists

Because general dentists regularly interact with patients, they are well positioned to screen for orofacial dysfunctions, speech disorders, sleep-disordered breathing, and poor oral hygiene. Close cooperation with speech-language pathologists is mandatory to restore proper orofacial functions, including optimal tongue posture and function, normal breathing and swallowing patterns, and improvement of speech. Maintaining good oral hygiene is essential to preventing tooth decay and extractions, both of which can complicate further orthodontic treatment. Working closely with an orthodontist who is experienced in treating patients with craniofacial disorders is crucial to providing the best possible treatment. A bi-retrognathic hyperdivergent facial pattern and growth deficits in both jaws are the presenting characteristics of patients with RS. Sagittal correction of the maxilla can be achieved through protraction treatment between six and nine years of age. Later, during the growth spurt, the growth and position of the mandible can be improved with functional orthodontics. In case of insufficient response to orthopedic treatment, orthognathic surgery may become necessary. Tooth extractions should be avoided before the growth spurt given the established role of each tooth in promoting jaw growth [44].

### 4.4. Strengths and Limitations

#### 4.4.1. Strengths

We present a homogeneous study population of individuals with RS that has been followed since birth. This population did not receive any treatments that modify facial growth during infancy, such as mandibular distraction osteogenesis, Tuebingen Palatal Plate placement, or orthognathic surgery during late adolescence. This series can be considered representative of the “natural history” of iRS. The orthodontic treatment consisted of only functional orthodontics, rapid maxillary expansion, and multibracket appliances, with tooth extraction therapy if necessary. None of our patients underwent orthognathic surgery, and only one had genioplasty. We used standard, high-reproducible orthodontic diagnostic tools. All measurements were carried out by the same experienced orthodontist. To our knowledge, this is the largest cohort studied since birth using a mixed-methods approach. Using multiple methods reduces the limitations associated with relying on one method. The same standard was used for documentation and evaluation for each patient. The same medical team provided follow-up care for this cohort.

#### 4.4.2. Limitations

When patients were born, CP was part of the diagnosis of RS in our institution. Thus, all patients presented CP. Therefore, it is unclear whether the maxillary deficiency is attributed to RS or CP repair. Although we included a large cohort of patients in our study, we could not analyze the cephalometric radiographs of some patients because of their poor quality. Metric analysis was not possible for all patients because some cephalometric radiographs lacked a measuring scale. We could include only a limited number of patients with Stickler syndrome, and only a few of them had available radiographs. Therefore, the results for these patients cannot be generalized and should be interpreted with caution. Although different practitioners performed the orthodontic treatment, all were specialists in orthodontics. Little information was available about the individual orthodontic treatments. The retrospective design of our study is an additional limiting factor.

## 5. Conclusions

RS seems to affect both jaws. During adolescence and with conservative orthodontic treatment, RS typically evolves into a harmonious aesthetic result with proportionate bimaxillary retrognathism, a normal jaw relationship (Class I), and a tendency toward a slight vertical facial pattern. Nevertheless, some of our patients presented skeletal Class III. Therefore, treatments associated with the risk of inducing skeletal Class III must be questioned. We demonstrated that the mandible partially catches up in growth during adolescence. The growth increments of the mandible in this period were greater than those of unaffected individuals. However, the mandible remained smaller in all dimensions. Nevertheless, this distinctive facial pattern predisposes patients with RS to obstructive sleep apnea. Patients with Stickler syndrome are at risk of severe bimaxillary retrognathism, midfacial hypoplasia, and a skeletal Class III pattern. For this reason, they require more intensive treatment. Of note, one-step versus two-step surgery for palatal repair and the degree of neonatal retrognathia did not affect skeletal sagittal parameters. Thus, the degree of neonatal retrognathia cannot be considered a prognostic factor for further mandibular growth. RS patients who initially experience severe functional problems are more likely to have a vertical (hyperdivergent) facial pattern. Additional research is needed to assess the impact of functional treatment on facial growth. These observations underscore the importance of functional treatment in this patient group. Overall, our cohort of adolescents with RS presented satisfactory facial appearances. Further studies should evaluate the impact of RS on the airway.

## Data Availability

The datasets analyzed during the current study are available from the corresponding author.

## Abbreviations

The following abbreviations are used in this manuscript:

ANOVA	Analysis of variance
CI	Confidence interval
CP	Cleft palate
CPAP	Continuous positive airway pressure
FH	Frankfort horizontal
ICC	Intraclass correlation coefficient
iCP	Isolated cleft palate
iRS	Isolated Robin sequence
OSA	Obstructive sleep apnea
QoL	Quality of life
RS	Robin sequence
SD	Standard deviation

## Appendix A

**Table A1:** Landmarks.

**Soft-tissue landmarks**	
G (glabella)	The most prominent anterior point in the mid-sagittal plane of the forehead.
N’ (soft-tissue nasion)	The point of the greatest concavity in the midline between the forehead and the nose.
Cm (columella)	The most posterior point of the lower border of the nose at which it begins to turn inferiorly to merge with the philtrum of the upper lip.
Sn (subnasale)	The point at which the columella (nasal septum) merges with the upper lip in the midsagittal plane.
Ls (labrale superius)	A point indicating the mucocutaneous border of the upper lip. Usually, the most anterior point of the upper lip.
Li (labrale inferius)	The median point on the lower margin of the lower membranous lip.
Sm (supramentale)	The most posterior midline point in the concavity of the chin.
Pog’ (soft-tissue pogonion)	The most prominent and anterior point on the chin in the midsagittal plane.
Me’ (soft-tissue menton)	Lowest point on the contour of the soft-tissue chin.
**Skeletal landmarks**	
S (sella)	The midpoint of the cavity of Sella turcica.
N (nasion)	The anterior point of the intersection between the nasal and frontal bones.
A (point A)	The innermost point on the contour of the premaxilla between the anterior nasal spine and the incisor tooth.
B (point B)	The innermost point on the contour of the mandible between the incisor tooth and the bony chin.
Pog (pogonion)	The most anterior point on the contour of the bony chin.
Gn (gnathion)	The most anterior–inferior point on the contour of the mandibular symphysis: right in the middle of the lower edge of the symphysis.
Me (menton)	The most inferior point on the mandibular symphysis (bottom of the chin).
Go (gonion)	A point on the curvature of the angle of the mandible located by bisecting the angle formed by lines tangent to the posterior ramus and the inferior border of the mandible.
Co (condylion)	The most superior and posterior point on the mandibular condyle.

**Table A2:** Angles.

**Soft-tissue angles**		
Facial convexity	GSnPog’	Angle defined by glabella-subnasale and soft-tissue pogonion.
Convexity of lower facial profile	Z angle	Angle defined by intersection of FH plane and a line connecting the soft-tissue pogonion and the most prominent lip.
Nasolabial angle	CmSnLs	Angle defined by columella, subnasale, and labrale superius.
Mentolabial angle	LiSmPog’	Angle defined by labrale inferius, supramentale, and soft-tissue pogonion.
Chin projection	LsN’Pog’	Angle defined by labrale superius, soft-tissue nasion, and soft-tissue pogonion.
**Skeletal angles**		
Anteroposterior position of the maxilla in relation to the cranial base	SNA	Angle defined by sella-nasion and point A.
Anteroposterior position of the mandible in relation to the cranial base	SNB	Angle defined by sella-nasion and point B.
Anteroposterior relationship between the maxilla and mandible	ANB	Angle defined by point A, nasion, and point B.
Facial plane divergence	SN-GoGn	Angle defined by the intersection of the anterior cranial base (SN) and the mandibular plane (GoGn).
FH plane–mandibular plane divergence	FMA	Angle defined by the intersection of the FH plane and the mandibular plane.
Gonial angle	CoGoMe	Angle defined by condylion, gonion, and menton.
